# Prognostic Value of AIMS65 Score in Cirrhotic Patients with Upper Gastrointestinal Bleeding

**DOI:** 10.1155/2014/787256

**Published:** 2014-12-22

**Authors:** Vinaya Gaduputi, Molham Abdulsamad, Hassan Tariq, Ahmed Rafeeq, Naeem Abbas, Kavitha Kumbum, Sridhar Chilimuri

**Affiliations:** Department of Medicine, Bronx Lebanon Hospital Center, 1650 Selwyn Avenue, Suite No. 10C, Bronx, NY 10457, USA

## Abstract

*Introduction*. Unlike Rockall scoring system, AIMS65 is based only on clinical and laboratory features. In this study we investigated the correlation between the AIMS65 score and Endoscopic Rockall score, in cirrhotic and noncirrhotic patients. *Methods*. This is a retrospective study of patients admitted with overt UGIB and undergoing esophagogastroduodenoscopy (EGD). AIMS65 and Rockall scores were calculated at the time of admission. We investigated the correlation between both scores along with stigmata of bleed seen on endoscopy. *Results*. A total of 1255 patients were studied. 152 patients were cirrhotic while 1103 patients were noncirrhotic. There was significant correlation between AIMS65 and Total Rockall scores in patients of both groups. There was significant correlation between AIMS65 score and Endoscopic Rockall score in noncirrhotics but not cirrhotics. AIMS65 scores in both cirrhotic and noncirrhotic groups were significantly higher in patients who died from UGIB than in patients who did not. *Conclusion*. We observed statistically significant correlation between AIMS65 score and length of hospitalization and mortality in noncirrhotic patients. We found that AIMS65 score paralleled the endoscopic grading of lesion causing UGIB in noncirrhotics. AIMS65 score correlated only with mortality but not the length of hospitalization or endoscopic stigmata of bleed in cirrhotics.

## 1. Introduction

Patients with acute upper gastrointestinal bleeding (UGIB) commonly present with either hematemesis or melena. The most important step in initial evaluation of such patients involves risk stratification with emphasis on assessing the hemodynamic status and its appropriate remediation. It is also imperative to examine the need for endoscopic intervention immediately after stabilization. There have been multiple scores that have been put forth to prognosticate the risk of rebleeding and inpatient mortality rate. These scores could be broadly divided into two categories: scores involving weighted endoscopic findings along with clinical and laboratory features (Rockall scores) and scores without the endoscopic criteria (AIMS65 and Blatchford scores). Katschinski et al. [1] proposed using Forrest classification, which predicts risk of rebleeding based on endoscopic stigmata, that actively spurting vessels have the highest chance of rebleed whereas clean based ulcer has the lowest. These predictors serve to help making an optimal choice towards endoscopic intervention directed towards achieving homeostasis (dual therapy versus single therapy versus no therapy).

The score proposed by Rockall et al. [2] incorporates both clinical, laboratory parameters like age, presence of shock, and comorbidities and endoscopic parameters including diagnosis and stigmata of bleeding. This score had been designed to predict the risk of continued bleeding and death. The score also therefore aids in determining the timing of endoscopic intervention. However, some studies have shown that the score is valid in predicting the risk of rebleed alone but not death [3]. Blatchford score [4] on the contrary takes into account only clinical criteria (hemoglobin, blood urea nitrogen, systolic blood pressure, pulse and the presence of melena, syncope, hepatic disease, or cardiac failure) but no endoscopic parameters. It has been shown that reliance on clinical parameters alone as is the case with Blatchford score does not affect its predictive value in determining the need for urgent therapeutic intervention [5]. It has also been shown by Blatchford et al. that this scoring system was better than Rockall score in predicting rebleeding and mortality as well [4]. AIMS65 score is scoring system that does not include endoscopic criteria and has been put forth as a good predictor of length of stay, cost of hospitalization, and mortality [6]. Out of the two common scoring systems not including endoscopic criteria AIMS65 outscored Blatchford score in predicting inpatient mortality from UGIB [7].

Clinical criteria included in computing these prediction scores often are confounded by existence of multiple coexisting comorbidities such as liver dysfunction and heart failure. Blatchford and Rockall scores include and give weightage to these comorbidities unlike AIMS65 score. Multiple studies have shown that AIMS65 singularly predicted few or all of the outcome measures in UGIB such as timing of endoscopy intervention, mortality, length of stay, and cost of hospitalization [8–10]. Preliminary data from Dekonda et al., comparing AIMS65 with preendoscopic Rockall score, showed that AIMS65 score was better at predicting the risk of rebleeding and inpatient mortality.

Patients with advanced liver disease often have hyperdynamic circulation with low blood pressure and systemic vascular resistance secondary to undermetabolized circulating vasodilators [11, 12]. Patients with advanced liver disease can also have impaired mentation from metabolic encephalopathy resulting from increased circulating levels of ammonia and gamma-aminobutyric acid (GABA) [13, 14]. Impaired synthetic function in advanced liver disease could manifest as decreased serum albumin and deranged coagulation profile (elevated prothrombin time). Therefore, theoretically AIMS65 scores could be elevated in patients with advanced liver disease at baseline. AIMS65 score does not provide additional scoring points to the presence of chronic liver disease. High mortality in patients with increased AIMS65 scores could therefore be a function of underlying severe liver disease itself, rather than that of UGIB.

In this retrospective study, we intended to see the correlation between the AIMS65 score and Endoscopic Rockall score. We looked at this correlation in cirrhotic patients and noncirrhotic patients separately. We wanted to examine the potential confounding role that underlying advanced liver disease could play while computing and interpreting AIMS65 scores.

## 2. Methods and Materials

### 2.1. Patients

This retrospective study was performed according to the Declaration of Helsinki with the Institution Review Board (IRB) approval at the study location. The period of study was five years from 2008 to 2012. Electronic medical records of all patients admitted to the hospital with overt UGIB who underwent esophagogastroduodenoscopy (EGD) were included in the study. The data was tabulated in Microsoft Excel. The patients who had minimal, self-limited UGIB not requiring a diagnostic or interventional endoscopic procedure were excluded from the study. The study patients were divided into two groups: patients with cirrhosis and patients without cirrhosis.

Baseline demographic data including age, gender, and ethnicity were collected for all patients in the study. AIMS65 scores were calculated at the time of admission. Endoscopic findings of each patient were reviewed and a weighted score was given as per Rockall Criteria. A separate weighted score for endoscopic stigmata of bleeding where applicable, as per Forrest classification (for ulcer bleeding only), was given as well. Etiologies of bleeding in cirrhotics and noncirrhotics (Table 1); etiologies of cirrhosis amongst patients included in the study (Table 2); and their baseline demographic, biochemical, and liver disease severity scores at the time of admission were collected (Table 3).

### 2.2. Evaluation of Results

We intended to see the correlation between AIMS65 scores and Endoscopic Rockall score in patient with and without cirrhosis separately. We also looked at these correlations separately in different genders and ethnicities.

### 2.3. Definitions

#### 2.3.1. Rockall Score [2]

The following variables were included for the computation of the score. The weightage given for each individual variable is included within the brackets:

- age [<60 years old (0 points), 60–79 years old (1 point), and ≥80 years old (2 points)];
- hemodynamic shock [systolic BP ≥ 100 mmHg and pulse < 100/min (0 points), tachycardic with pulse ≥ 100/min but systolic BP ≥ 100 mmHg (1 point), and hypotension with systolic BP < 100 mmHg (2 points)];
- major comorbidities [none (0 points); cardiac failure, ischemic heart disease, or similar major comorbidity (2 points); renal failure, hepatic failure, or disseminated cancer (3 points)];
- diagnosis on endoscopy [Mallory-Weiss tear but no major lesions and no stigmata of recent bleed (0 points), other nonmalignant gastrointestinal diagnoses (1 point), and upper gastrointestinal tract malignancy (2 points)];
- stigmata of recent hemorrhage [none (or dark area only) (0 points), blood found in upper gastrointestinal tract (clot adherence, spurting, or visible vessel) (2 points)].

A score of 2 or less was shown to be associated with a low risk of further bleeding or death.

#### 2.3.2. AIMS65 Score [6]

The following variables were included for the computation of the score. The weightage given for each individual variable is included within the brackets:

- albumin (1 point for value less than 3.0 g/dL (30 g/L));
- INR (1 point for value greater than 1.5);
- altered mental status (1 point given if Glasgow coma score was less than 14 or if disorientation, lethargy, stupor, or coma was seen);
- systolic blood pressure (1 point for value less than 90 mmHg);
- age (1 point for value greater than 65 years).

The validation cohort showed that the mortality risk increased from 0.3% in patients with AIMS65 score of 0 to almost 25% in patients with AIMS65 score of 5.

#### 2.3.3. Forrest Classification [1]

The classification was based on stigmata of bleed seen on endoscopy. The weightage given for each individual variable is included within the brackets:

- active arterial bleeding (Forrest Ia—score of 5), oozing without visible vessel (Forrest Ib—score of 4);
- nonbleeding visible vessel (Forrest IIa—score of 3), adherent clot (Forrest IIb—score of 2), and flat spot (Forrest IIc—score of 1);
- clean ulcer base (Forrest III—score of 0).

### 2.4. Statistical Analysis

Continuous variables were stated as mean and standard deviation. Unpaired* t*-test was used for comparison of means. A* P* value < 0.05 was considered statistically significant. Linear regression was used to look at the relationship between two continuous variables.

## 3. Results

**Figure 1:**
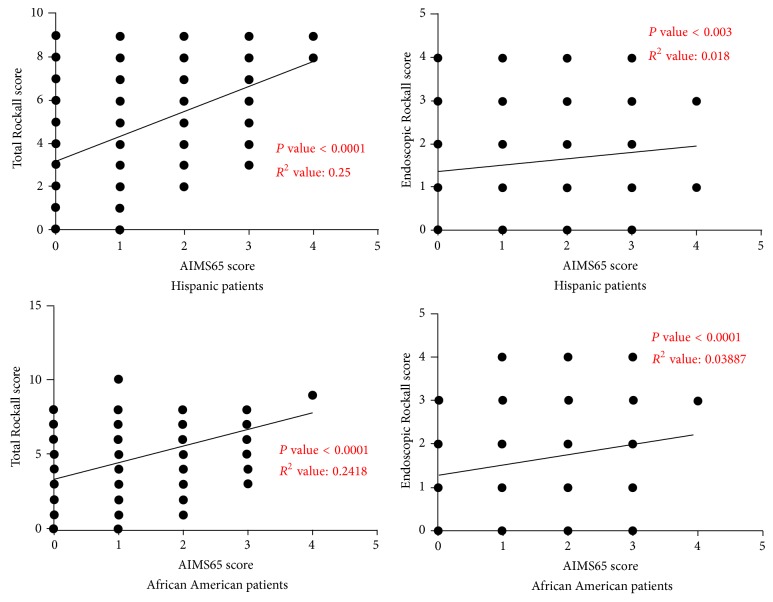
Statistically significant correlation was found between Total Rockall/Endoscopic Rockall scores and AIMS65 scores in both Hispanic and African American patients.

A total of 1255 patients with diagnosis of overt UGIB presented to the hospital and underwent EGD during the study period between January 2008 and December 2012. Out of the study population, 152 patients were found to have cirrhosis while the remaining 1103 patients were without cirrhosis. Out of the study population 761 patients were male while 494 were female. There were 719 Hispanics, 484 African Americans, 45 Caucasians, 4 Asians, and 3 people of Indian origin in the study population. We looked at the correlation of AIMS65 score with Total Rockall score and Endoscopic Rockall score separately within each ethnicity (Figure 1). Statistically significant correlation was found between Total Rockall/Endoscopic Rockall scores and AIMS65 scores in both Hispanic and African American patients.

**Figure 2:**
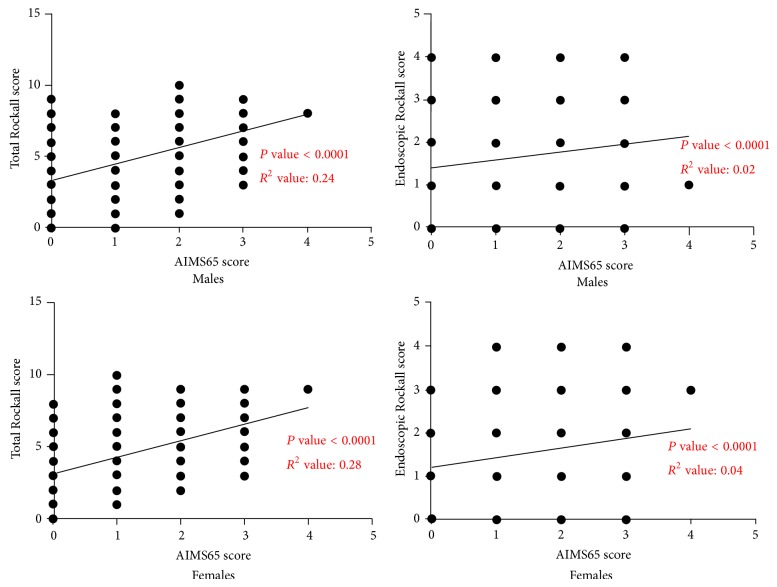
Statistically significant correlation was found between Total Rockall/Endoscopic Rockall scores and AIMS65 scores in both genders.

We also computed the correlation of AIMS65 score with Total Rockall score and Endoscopic Rockall score separately within each gender (Figure 2). Statistically significant correlation was found between Total Rockall/Endoscopic Rockall scores and AIMS65 scores in both genders.

**Figure 3:**
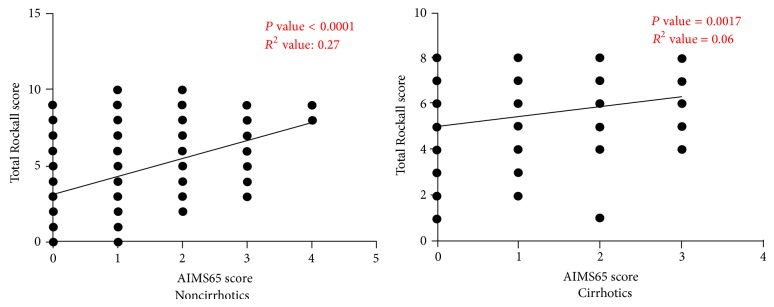
Statistically significant correlation was found between Total Rockall scores and AIMS65 scores in both noncirrhotic and cirrhotic patients.

We also found that there was significant correlation between AIMS65 score and Total Rockall score in patients both without and with cirrhosis (Figure 3).

**Figure 4:**
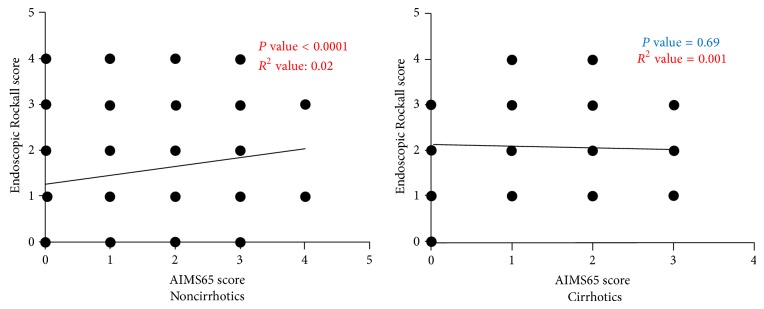
Statistically significant correlation was found between Endoscopic Rockall scores and AIMS65 scores in noncirrhotic patients but not cirrhotic patients (*P* value = 0.69).

We performed correlation analysis between AIMS65 score and Endoscopic Rockall score in patients without cirrhosis and with cirrhosis separately (Figure 4). We found that there was significant correlation between these variables in only noncirrhotics but not in patients with cirrhosis.

**Figure 5:**
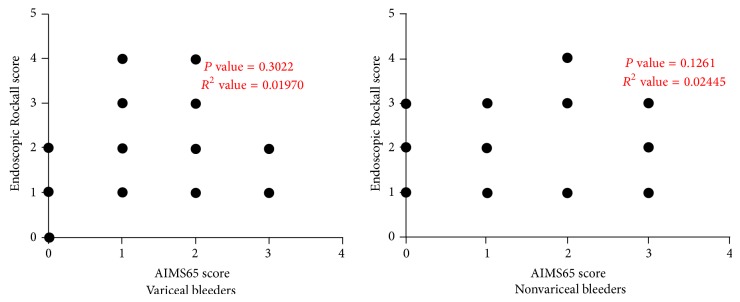
No significant correlation was found between Endoscopic Rockall scores and AIMS65 scores in both cirrhotic variceal (*P* value = 0.3022) and nonvariceal bleeders (*P* value = 0.1261).

Out of the 152 patients with cirrhosis, 96 patients had nonvariceal bleed whereas 56 had variceal bleed. There was no significant correlation between AIMS65 scores and Endoscopic Rockall scores in either of these groups (Figure 5).

**Figure 6:**
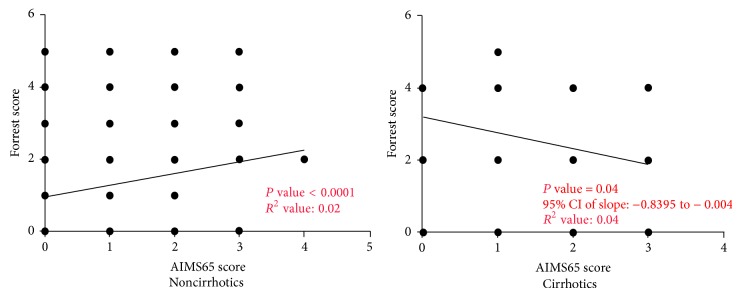
Statistically significant direct correlation was found between weighted Forrest scores and AIMS65 scores in noncirrhotic patients. Statistically significant inverse correlation was found between weighted Forrest scores and AIMS65 scores in cirrhotic patients.

There were 715 patients in the study group (618 patients without cirrhosis and 97 with cirrhosis) who were diagnosed with UGIB secondary to peptic ulcer disease. The endoscopic stigmata of bleeding were given weighted scores as per Forrest Criteria. We performed correlation analysis between AIMS65 score and the weighted Forrest Criteria scores in patients without cirrhosis and with cirrhosis separately. There was a statistically significant correlation between the AIMS65 score and weighted Forrest score in noncirrhotics, but it was not the case in cirrhotics; no such correlation was found (Figure 6).

**Figure 7:**
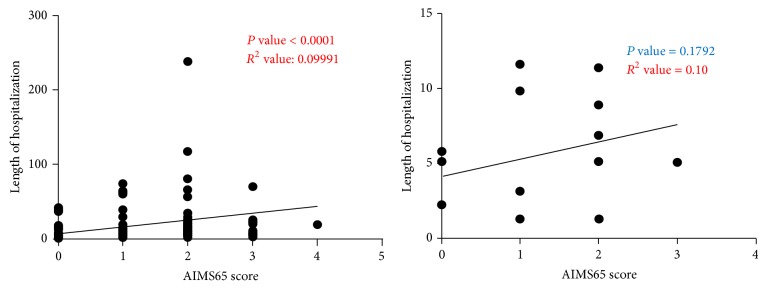
Statistically significant direct correlation was found between the length of hospitalization and AIMS65 scores in noncirrhotic patients but not cirrhotic patients.

Out of the total study population of 1255 patients, only 160 (141 noncirrhotics and 19 cirrhotics) patients required admission. We examined the correlation between the AIMS65 score and length of stay in patients admitted with UGIB. There was significant correlation between these two variables in noncirrhotic patients (*P* value < 0.0001) but not in cirrhotic patients (*P* value = 0.1792) (Figure 7).

**Figure 8:**
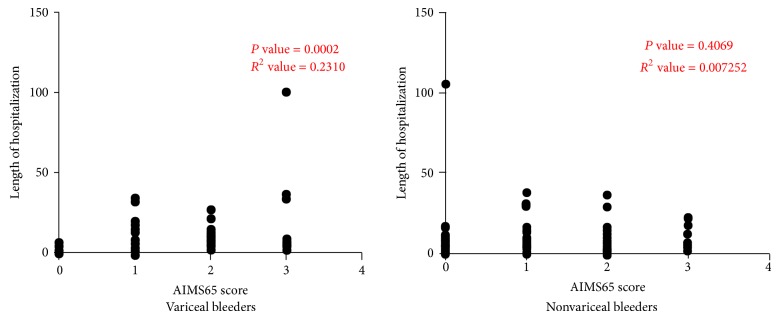
Statistically significant direct correlation was found between the length of hospitalization and AIMS65 scores in cirrhotic patients with variceal bleed but not in cirrhotic patients with nonvariceal bleed.

There was a significant correlation between the length of hospitalization and AIMS65 scores in cirrhotic patients with variceal bleed (*P* value = 0.0002) but not in cirrhotic patients with nonvariceal bleed (*P* value = 0.4069) (Figure 8).

**Figure 9:**
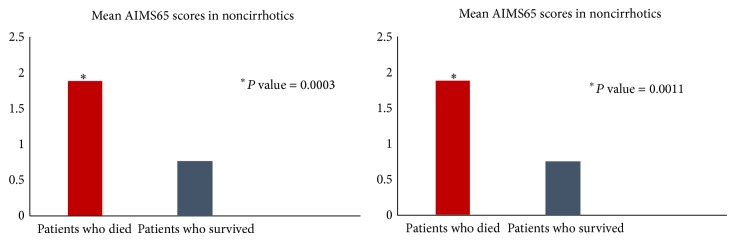
Mean AIMS65 scores were significantly higher in patients who died than in patients who survived, in both noncirrhotic and cirrhotic groups.

There were a total of 15 deaths within the study group with 8 in noncirrhotic and 7 in cirrhotic group, respectively. The AIMS65 score in the noncirrhotic group was significantly higher in patients who died from UGIB (mean AIMS65 score: 2 ± 0.75) than in patients who did not (mean AIMS65 score: 0.80 ± 0.85) (*P* value = 0.0003). Similarly in the cirrhotic group, average AIMS65 score in patients who died from UGIB (mean AIMS65 score: 2.14 ± 0.37) was significantly higher than that of patients who did not (mean AIMS65 score: 1.08 ± 0.82) (*P* value = 0.0011) (Figure 9).

## 4. Discussion

AIMS65 score has been shown to predict mortality in multiple studies [7, 9]; however the value of AIMS65 score in predicting the need for endoscopic intervention has not been proven [9]. We wanted to examine the prognostic value of AIMS65 score in patients with cirrhosis presenting with UGIB. Individual components used for calculation of AIMS65 score (barring age) are greatly influenced by underlying liver disease. This is evident from the observation that there was a direct correlation between the AIM65 score and MELD score in patients admitted to the hospital with UGIB. We also found a direct correlation between Total Rockall score and AIMS65 score in both cirrhotics and noncirrhotics alike, as there is a certain overlap of variables used in the calculation of respective scores (age, hypotension). Remarkably we also found a direct correlation between AIMS65 score and endoscopic scores (Endoscopic Rockall score and Forrest score) in noncirrhotics but not in cirrhotics. Our data therefore shows that AIMS65 score is a poor predictor of endoscopic finding in cirrhosis. There was no correlation between AIMS65 score and length of hospital stay in cirrhotics with nonvariceal bleeding. However, we did find a significant correlation between AIMS65 score and length of hospital stay in cirrhotics with variceal bleeding. However this increased length of stay could be a function of MELD score (and therefore the severity of underlying intrinsic liver disease) rather than AIMS65 score itself.

Our study was beset by disadvantages inherent to a retrospective study. Even though we took only patients with UGIB as primary admitting diagnosis, possible multiple unrecognized confounding variables could have influenced the lengths of hospitalization. The contribution of underlying intrinsic liver disease and other medical comorbidities (including congestive heart failure and ischemic heart disease which are accounted for, in calculation of Total Rockall score) to the length of hospitalization could not be individually ascertained. The system of grading endoscopic stigmata using Forrest Criteria is partly subjective and has potential for significant interobserver variability. AIMS65 score has been shown to predict the need for endoscopic intervention in patients with UGIB. We only included patients who had an endoscopic intervention in our study. This could have biased our results significantly, as performing an endoscopic intervention in itself could have contributed to lengthening the hospital stay.

In conclusion, we observed statistically significant correlation between AIMS65 score and length of hospitalization as well as mortality in noncirrhotic patients. We also found that AIMS65 score paralleled the endoscopic grading of lesion causing UGIB in such patients. In cirrhotic patients, AIMS65 score correlated only with mortality but not endoscopic stigmata of bleed (in nonvariceal bleeders). AIMS65 score correlated with length of hospitalization in variceal bleeders but not cirrhotic nonvariceal bleeders. AIMS65 score, computation of which is influenced by underlying liver disease, is not a strong predictor for severity of gastrointestinal lesion on endoscopy or length of hospitalization in cirrhotics. We cannot make a comment on value of AIMS65 score in determining the need for endoscopic intervention (even as AIMS65 score poorly correlated with endoscopic findings), as we designed the study to include only patients who underwent endoscopy. This is especially true in cirrhotics who present with UGIB where a simplified preendoscopy risk stratification system that predicts necessity of endoscopy and hospitalization is often irrelevant as almost all patients are hospitalized and undergo urgent diagnostic or therapeutic endoscopy. However, AIMS65 score may be predictive of mortality and postendoscopic clinical course in cirrhotics including length of hospitalization. In view of the limitations of this study, further investigation is needed to clearly elucidate the role of scoring systems such as AIMS65 in predicting clinical outcomes in patients with cirrhosis.

## Figures and Tables

**Table 1:** Depiction of etiologies of bleeding in cirrhotics and noncirrhotics, respectively.

Etiology of bleeding	Cirrhotics	Noncirrhotics	*P* value
Esophageal varices (%)	56 (36.8)	NA	NA
Peptic ulcer disease (%)	71 (46)	553 (50.1)	0.4375
Esophagitis (%)	20 (13.1)	426 (38.6)	0.0001^*^
Mallory-Weiss tear (%)	5 (3.3)	124 (11.2)	0.0015^*^

^*^ *P* value < 0.05 was considered statistically significant.

**Table 2:** Distribution of etiologies of cirrhosis among study subjects.

Etiology of liver cirrhosis	Number (%)
Hepatitis-C	107 (70.4)
ETOH	43 (28.3)
Cryptogenic (probably NAFLD^*^)	2 (1.3)

^*^Nonalcoholic fatty liver disease.

**Table 3:** Baseline demographic, biochemical, and liver disease severity scores among cirrhotic patients.

Variable	Mean (±SD^§^)	
Age	54.78 (12.95)	
INR^*α*^	1.41 (0.49)	
Albumin	2.76 (0.79)	
Serum creatinine	1.83 (1.87)	
Total serum bilirubin	3.11 (2.99)	
AST^*π*^	93.97 (110.03)	
ALT^¥^	65.66 (105.22)	
MELD score^€^	10.09 (4.54)	
MELD score in variceal bleeders	9.05 (4.39)	*P* value^*^ = 0.006
MELD score in nonvariceal bleeders	10.7 (2.9)	

^§^Standard deviation.

^*α*^International normalized ratio.

^*π*^Aspartate aminotransferase.

^¥^Alanine aminotransferase.

^€^Model for end-stage liver disease.

^*^ *P* value < 0.05 was considered statistically significant.
